# A debate on current eating disorder diagnoses in light of neurobiological findings: is it time for a spectrum model?

**DOI:** 10.1186/1471-244X-12-76

**Published:** 2012-07-06

**Authors:** Samantha Jane Brooks, Mathias Rask-Andersen, Christian Benedict, Helgi Birgir Schiöth

**Affiliations:** 1 Department of Neuroscience, University of Uppsala, Box 593, Uppsala, Sweden

**Keywords:** DSM, Anorexia, Bulimia, Binge-eating, Genetic, fMRI

## Abstract

**Background:**

Sixty percent of eating disorders do not meet criteria for anorexia- or bulimia nervosa, as defined by the Diagnostic and Statistical Manual version 4 (DSM-IV). Instead they are diagnosed as ‘eating disorders not otherwise specified’ (EDNOS). Discrepancies between criteria and clinical reality currently hampering eating disorder diagnoses in the DSM-IV will be addressed by the forthcoming DSM-V. However, future diagnoses for eating disorders will rely on current advances in the fields of neuroimaging and genetics for classification of symptoms that will ultimately improve treatment.

**Discussion:**

Here we debate the classification issues, and discuss how brain imaging and genetic discoveries might be interwoven into a model of eating disorders to provide better classification and treatment. The debate concerns: a) current issues in the classification of eating disorders in the DSM-IV, b) changes proposed for DSM-V, c) neuroimaging eating disorder research and d) genetic eating disorder research.

**Summary:**

We outline a novel evidence-based ‘impulse control’ spectrum model of eating disorders. A model of eating disorders is proposed that will aid future diagnosis of symptoms, coinciding with contemporary suggestions by clinicians and the proposed changes due to be published in the DSM-V.

## Background

Despite obvious physical and behavioral signs, up to sixty percent of referrals for eating disorders (ED), are not given a specific diagnosis, but instead labeled with Eating Disorder Not Otherwise Specified (EDNOS) [1-3]. Anorexia Nervosa (AN) and Bulimia Nervosa (BN) are the specific ED diagnoses available in the Diagnostic and Statistical Manual of Mental Disorders version 4 (DSM-IV), currently being revised for a fifth edition due to be released in May 2013 [4]. AN and BN are recognizable by severe emaciation and uncontrolled eating patterns respectively, but these indicators are not enough for diagnosis following the current criteria. Additionally, despite the presence of neuropsychological disturbances in people with ED being known (e.g. ruminations and obsessions about weight, shape and eating), they are merely eluded to in the current diagnostic criteria (e.g. fear of weight gain and a sense of lacking control). The diagnostic machinery and treatment responses that rely heavily on the DSM-IV ED criteria to assess improvement in symptoms will be re-oiled in 2013 following the publication of the DSM-V, which plans to loosen the boundaries in line with a more transdiagnostic approach. This paradigm shift has been undoubtedly buoyed by the continuing advances in our understanding of the underlying neurobiological mechanisms of disordered eating, through genetic studies, and illustrated using technologies that measure brain structure and function. In this debate article, we will briefly summarize the current DSM-IV criteria underlying the ED diagnoses; we will then encapsulate findings from brain imaging and genetic studies in people with ED, against the background of the old and new diagnostic systems. We finish by proposing an “impulse control” spectrum model of eating behavior that may help to better visualize symptom thresholds that cross the normalcy boundaries and into eating disorder.

Eating disorders (ED) which, by definition in the DSM-IV constitute anorexia nervosa (AN) and bulimia nervosa (BN) are typically female adolescent-onset psychiatric conditions. A third type, binge eating disorder (BED) is informally mentioned in the Appendix of DSM-IV, but is set to be included as a third ED in the forthcoming DSM-V. Current lifetime prevalence rates using screening and diagnostic measures of AN and BN for females in Western populations are estimated to be approximately 0.3 and 0.9 percent respectively [5], for males the prevalence rate has been reported as 0.03 percent, but data is only available for AN [6]. Estimates based on epidemiological measures are slightly higher for females, at 0.7 percent for AN and 2 percent for BN females. Current DSM-IV diagnostic criteria for EDs are narrowly defined, specific to female adults and subject to alterations that will be set out in the imminently published DSM-V. Diagnosing a specific ED is further complicated by other major psychiatric conditions that share similar clinical symptoms, such as anxiety and depression [7].

Sufferers of AN are most identifiable by their severe emaciation, cognitive disturbances and continued refusal to eat. Presently, a formal diagnosis of AN must satisfy three DSM-IV criteria: refusal to maintain body weight at or above the expected minimum, thus weighing 85 percent or less of expected body weight or a Body Mass Index (BMI, kg/m^2^) of ≤17.5; possessing an intense, pathological fear of gaining weight; and amenorrhea (lack of menses) for three consecutive months not related to any other medical condition. Recommendations for amendments to the DSM-V include that the word *refusal* be removed as a potentially pejorative term that is difficult to ascertain clinically [8]. Furthermore, it is difficult to measure intense fear, particularly in those who deny fear, and so it is suggested that an extra clause be added to address this [8,9]. Finally, it is proposed that amenorrhea be deleted from the formal diagnostic criteria in DSM-V to account for pre-pubescent female sufferers, and the small percentage of males who reportedly suffer [10]. Within the main diagnosis of AN, two subtypes are recognized by the DSM-IV that differ in the amount of food consumed: restricting and binge purging AN. The former is the most severe, whereby sufferers consume only morsels of food often with fatal consequences if left untreated, whereas in the latter sufferers eat relatively more yet are still emaciated due to the adoption of compensatory measures to reduce weight (e.g. vomiting and laxative abuse). However, due to much ‘cross-over’ between the subtypes in the ‘current episode’ of illness it has been suggested that for DSM-V, in line with the timeframe used for the diagnosis of BN, that the subtype behavior (e.g. restricting versus bingeing) be consistently present for the last three months [11]. In both subtypes, sufferers exhibit severe cognitive disturbances. These include; excessive perfectionism, asceticism [12], cognitive rigidity and deficits in set-shifting (concrete adherence to rigid rules rather than being able to adjust to changing rules) which also pertains to excessive attention to detail [13], and ruminations, obsessions about food and excessive concerns about weight and shape [14]. These cognitive deficits are often present during adolescence before the onset of eating disorder, and can remain following weight restoration in recovery, and are regarded by some as potential indicators of risk for developing an ED [15].

To be diagnosed with BN, the DSM-IV criteria are: recurrent episodes of bingeing and purging must occur at least twice weekly over a period of three months. A binge constitutes the consumption of an amount of food that most people would consider to be large, whilst lacking control over eating over a short period of time (e.g. half an hour). Purging includes compensatory behaviors such as vomiting, laxative abuse, excessive exercise or intermittent food restriction to counteract weight gain. However, a recent review showed that bulimic symptoms were still largely present in individuals reporting a lower frequency (e.g. once per week) of binges and purges [16], and so it is likely that for the DSM-V the frequency will be lowered to once per week over a three month period. Currently, the DSM-IV recognizes two subtypes of BN: purging and non-purging, the former requires that compensatory measures occur intermittently between binge episodes, whereas the latter does not require purging to occur, but instead bouts of restricted eating (which differs from AN due to the lack of emaciation in BN). However, according to one recent review, it is currently unclear how to precisely define non-purging behavior [17], and thus, it is suggested that the subtypes be deleted from the BN definition for DSM-V.

BED is not currently recognized as a formal diagnosis of ED by the DSM-IV but is instead included in the Appendix. However, a list of reasons are highlighted based on a recent review [18] that support the suggestion for BED to be formally recognized as a third type of ED in the DSM-V. One of the main reasons is that the dysfunctional eating behaviors underlying BED have been compared to both AN and BN (e.g. fear of weight gain, lack of control over eating). In terms of ED antecedents there is evidence that BED has a strong familial history component and is not simply a form of obesity (which is not currently recognized as an ED in the DSM-IV, nor suggested as an ED in DSM-V). Furthermore, BED has a distinct demographic profile in that it is predominantly found in males with a later age of onset to other EDs. It is also plausible that BED can be included as an ED from the range of psychological disturbances observed in sufferers (which are thought to be mostly absent in those who are obese). For example, excessive concerns about weight and shape, personality disturbance and psychiatric comorbidities in the form of mood and anxiety disorders, combined with a lower quality of life. However, the difficulties in recognizing BED as a formal ED include; a lack of diagnostic stability and high levels of remission. Additionally, BED coincides with medical morbidities that are not observed in other EDs, for example the incidence of metabolic syndrome (e.g. hypertension, type 2 diabetes). However, it has been shown that highly specific treatments are effective for BED [18], supporting the clinical relevance of recognizing it as a third ED. Thus, it is proposed, in line with the criteria for BN, that BED be defined by recurrent episodes of binge eating (e.g. at least one per week for three months), combined with a sense of lack of control over eating, and without inappropriate compensatory behaviors (e.g. purging, excessive exercise).

Those who exhibit pathological disordered eating behaviors but who do not meet the full criteria for AN or BN as stipulated in the DSM-IV described above, are instead diagnosed as ‘*Eating Disorder Not Otherwise Specified’* (EDNOS). According to recent reports the diagnosis of EDNOS, which is marked by cognitive dysfunction and suboptimal functioning is given to up to sixty percent of cases presenting to medical professionals [1-3]. This is problematic, since it has been shown that reduction of ‘psychological dysfunction’ is linked to lower relapse rates than recovery that is defined by the absence of DSM-IV criteria, e.g. weight and menses restoration [19]. Thus, there are some major issues in the current DSM-IV that prevent an effective diagnosis and subsequent treatment. These include males, pre-pubescent girls, and adolescent females with regular menses who are engaged in infrequent bingeing and purging, whose body weight exceeds the 85^th^ percentile expected for their age but do not have healthy eating patterns (i.e. are persistently worried about their weight, shape and eating), those who deny, or for whom it is difficult to measure a pathological fear of gaining weight. These discrepancies are an obvious mismatch of the current diagnostic system to clinical reality [3] in that patients assigned to the EDNOS category are not adequately supported by treatments currently tailored to a definite diagnosis of AN and BN as emphasized by the current DSM-IV. These treatments include: a) Cognitive Behavioral Therapy (CBT) that counters dysfunctional cognitions, b) psychodynamic approaches that address ‘hysterical’ psychological conflicts and conversion disorders, c) pharmacological interventions such as Selective Serotonin Reuptake Inhibitors (SSRIs) or low-dose second generation anti-psychotics that tackle neurotransmitter imbalances in the brain. It has been suggested that the EDNOS category could be diminished to represent a mere eight percent of cases (instead of 60 percent) with new diagnostic labels of ‘purging disorder’ [20] and ‘restrained eating disorder’ [3], which meet some but not all of the extreme criteria for BN and AN respectively.

To introduce contemporary neurobiological knowledge of eating disorders in to the debate on how to best improve classification of eating disorders, we will next highlight conclusions from neuroimaging and genetic research. By no means do we present an exhaustive meta-analysis or review of the literature, but rather, set out some recent findings and popular contemporary views of the neurobiology of eating disorders. Finally, we propose how these findings might be incorporated in to an “impulse control” model of eating disorders, such that we prompt a debate on what distinguishes separate eating disorders, and whether it is plausible to include them on a single spectrum.

## Discussion

### Brain imaging research in to disordered eating behaviour

This section summarizes conclusions drawn from research involving functional brain imaging methods, that provide some evidence to support a neural model of eating disorders across a spectrum of restricted versus impulsive eating behavior. It is not meant to be an exhaustive meta-analytical review, which has been done by expert researchers in the field previously (see below) but a summary of the main views arising from contemporary brain imaging findings, in an attempt to spark a debate about a model of disordered eating behaviour.

As has been eloquently reviewed previously [21-24], functional brain imaging studies of females with AN (in comparison to healthy, age and gender matched controls), particularly when viewing rewarding stimuli, largely report aberrant, often reduced activation in ‘bottom-up’ mesolimbic regions associated with somatic states (e.g. appetite) such as in the striatum, hippocampus, amygdala, hypothalamus and cerebellum. This is often in conjunction with increased activation in ‘top-down’ prefrontal cortical (PFC) regions linked to cognitive evaluation, attention and executive functioning (e.g. working memory, goal-orientation, self-reference, evaluation of salience) such as the dorsolateral prefrontal cortex (DLPFC), medial prefrontal cortex (mPFC), orbitofrontal cortex (OFC), and anterior cingulate cortex (ACC). Furthermore, there appears to be a strong indication from various neuroimaging modalities (e.g. Positron Emission Tomography, Single Photon Emission Tomography) that the subtypes of AN (e.g. restricting and binge purging) can be distinguished by relatively higher or lower activations to appetitive stimuli in these regions [25]. Indeed other fMRI studies show that the DLPFC, which appears to be highly activated when thinking about eating food shown in images in people with AN [26,27], is strongly implicated in both appetite suppression and working memory function [28-30]. Also, a recent neuropsychological study showed that automatic appetite activation (using subliminal images of food) interfered with a DLPFC-dependent working memory task [31]. Thus, the excessive ruminations about weight, shape and eating in those who pathologically restrain their appetite is reminiscent of classic, yet excessive, working memory function. In turn, the ability to restrain appetite in those with anorexia might be associated with excessive top-down activation (mainly DLPFC) to varying degrees (e.g. different levels of restraint between the subtypes of AN), combined with varying levels of desire to restrain an over or underactive appetitive system. Furthermore, recent evidence suggests that the neural response during working memory performance is modulated by genetic polymorphisms in COMT and BDNF described below [32]. However, there is one caveat to linking hyperactivation in the DLPFC to excessive working memory function in AN: it is currently unclear whether increased DLPFC working memory-related activation reflects a deficiency in working memory and a need to work harder.

**Figure 1:**
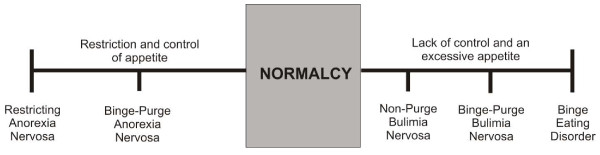
Impulse-control spectrum model of eating disorders.

Conversely, in those who exhibit binge eating behavior (e.g. BN, BED), previous research summarises a neural pattern to rewarding stimuli that is somewhat reversed, with a reduced or sporadic response in PFC regions, combined with hyperactivation in mesolimbic areas. In further support, a recent fMRI study that directly compared neural activation to food images in women with AN and BN found a clear PFC vs. striatum response to images of food [33]. However, it is also plausible that binge eating might coincide with hypofunctioning of the reward system [34], prompting the hypothesis that during binge eating, like an addict, a person must consume greater quantities of food to achieve the feeling of satiation. Furthermore, there appears to be a differential pattern of activation in binge eaters, when consuming versus anticipating food consumption during fMRI (e.g. [35]), suggesting that the model proposed here might be more applicable to anticipation of food intake, and cognitive biases towards food stimuli. It is also often observed that people who binge (e.g. those with BN) have a strong desire to restrain their intake, and recent evidence suggests that restraining one’s appetite increases the chances of binge eating in those with a genetic risk for binge eating [36]. Moreover, recent evidence suggests that artificial stimulation of the DLPFC using repetitive Transcranial Magnetic Stimuluation (rTMS) reduces craving in those prone to binge-eat [37]. Thus, the neurobiological interactions underpinning restrained and binge eating behaviour are complex. It is proposed here that, in response to appetitive food stimuli that may underlie responses to food, a specific neural signature, with sporadic dominance of restraint versus binge eating can be mapped onto a single model with the support of recent neuroimaging findings. See Figure 1.

**Figure 2:**
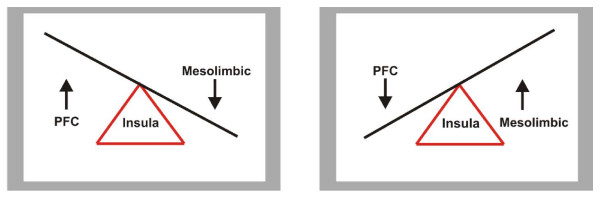
Imbalanced PFC-mesolimbic convergence on the insular cortex in those with eating disorders.

A commonality between all ED subtypes is seemingly dysregulated neural activation in response to food stimuli in the parietal cortex, and the insular cortex in the temporal lobe, regions associated with somatosensory perception and interoceptive awareness [25,38]. One might argue that somatosensory perception occurs via a balance between basic bottom-up arousal systems, and higher order top-down cognitive evaluations. Against this background, it is proposed that an excessive activation at one or other extremes (e.g. restraint versus binge eating systems) in those with eating disorders causes an imbalanced convergence on these somatosensory brain regions, associated with dysfunctional processing of the body state (inexorably linked to body image distortions). See Figure 2. Additionally, hyperactivation of the amygdala is often seen across ED diagnoses [39,40] and likely leads to anxiety experienced by most ED sufferers regardless of subtype. Emotional experiences that derive from somatosensory states can be positively or negatively arousing (e.g. pleasant feelings during appetite satisfaction versus unpleasant anxiety and anger) and are linked to areas of the ‘temporoparietal junction’ that are involved in creating a sense of self-control over one’s actions [41]. It has long been established that people with ED have problems identifying and expressing their emotions [42], a core symptom known as *alexithymia*, which has been further demonstrated in recent neuropsychological studies [43,44]. An imbalanced regulatory neural circuit between the PFC and mesolimbic regions likely hampers a healthy orchestration of these systems, leading to a ‘rate-limiting’ defect in the insula [45] and other somatosensory regions that normally provide a solid sense of ‘emotional self’ (See Figure 2).

Findings from fMRI research into obesity, which could be regarded as representing the excessive appetitive extreme of the proposed spectrum model of eating disorders, fit well with the notions proposed here. One might predict, against the background of the present taxonomy, that people who are obese have deficits in prefrontal cortical activation, particularly the DLPFC, combined with hyperactivation of brain reward systems. Such a neural substrate does appear to exist in obesity, in that a hypersensitive reward system is demonstrated, in response to various types of food stimuli [46], but particularly when overweight people think about the taste of food [47]. Hyperactivation of reward systems when thinking of and/or viewing food stimuli may well impinge on cognitive systems, particularly memory and cognitive control, in those who are obese [46]. However, causality is difficult to determine, and it could also be that deficits in cognitive processing prevent an adequate top-down control of one’s impulsive responses, e.g. over-eating in those who are obese. Thus, fMRI studies of obesity do seem to support the notion that specific eating disorder symptoms, ranging from restraint to impulsivity, can be mapped on to a single spectrum model, as proposed here.

Eating disorder cases that do not easily fit in to the proposed model must also be considered in this debate. For example, *prima facie* it is not easy to fit binge-purge anorexia nervosa to the model, because such cases often consume excessive amounts of food in one sitting, while subsequently adopting compensatory measures (e.g. vomiting, laxative abuse) to avoid weight gain, and so might not easily fit the restrictive extreme. Furthermore, profile analyses of individual diagnoses of eating disorders often exhibit an inhibited and non-inhibited style based on personality traits [48,49], suggesting that disordered eating behaviour is based not on appetitive and restricting temperaments, but more on personality dimensions. However, we argue that the model can also explain these cases, because we do not suggest that a person is exclusively *restrictive* or *impulsive*. Rather, we purport that, depending on one’s response to fluctuating social/environmental challenges, at any given time a person can present as restrictive or impulsive. Against this background, we further suggest that it is *temperamental dominance* that dictates which behaviour is more frequently observed (restrictive vs. impulsive), for example, a person with AN who is generally restrictive, but with bouts of binging and purging. Temperamental dominance might also underlie personality characteristics, e.g. a person with a restrained eating disorder might also be introverted and mildly psychotic, a person with an impulsive eating disorder might present features of extroversion and neuroticism. Temperamental dominance, we purport, is determined by inherent neurobiological factors that are acquired genetically, but modified epigenetically via learned interactions with new environmental challenges (e.g. during puberty), particularly in those who develop eating disorders [50].

It is of note that despite its apparent appeal, body weight may not be the most accurate measure to indicate one’s position on the spectrum, because for example, some people with bulimia are able to excessively exercise to compensate for high levels of food consumption - a factor that makes bulimia nervosa difficult to detect and treat. Furthermore, cognitive and personality traits often precede starvation and are sometimes present following weight restoration. It could be that questionnaire measures of temperament (e.g. Restraint Scale [51], Eysenck Impulsivity Questionnaire [52]) will prove more appropriate for measuring susceptibility or current diagnosis of disordered eating behaviour at a given timepoint. Thus, in the proposed spectrum model, a fluctuating temperament linked to eating could present (e.g. restraint, binge-purge, extroversion-introversion), but it is the temperamental dominance, persisting over time (e.g. excessive restriction, excessive food consumption, regardless of compensatory measures) that determines ones true place on the model.

Another major caveat to the proposed model are the recent findings that self-starvation may evoke reward responses in the brain [53,54]. In line with animal models of AN, this suggests that the stress induced by self-starvation becomes a rewarding and/or motivating factor. However, despite fMRI studies showing increased striatal responses in females with AN, it is to images of thin bodies (which are probably rewarding for a person with AN to look at, relative to larger bodies). Previous fMRI studies using images of bodies (e.g. [55-57] show different neural responses to food stimuli, and appetitive activation is the main focus of the model proposed here. Furthermore, it could be that the striatal response to self-starvation does not reflect reward, but rather an increased motivational state (which could explain a greater propensity to exercise in AN, despite energy deficits), akin to the evolutionary homeostatic drive to seek food [58]. Given that the motivation neural circuity, although shown to be separate, is closely tied to reward responses in the mesolimbic pathway (e.g. [59]), it might be difficult to separate food seeking behaviour from pleasurable responses to food in those with AN. The problem of the model therein lies: how to differentiate between striatal responses associated with seeking, and striatal responses associated with impulsive and excessive binge eating. In an attempt to account for this discrepancy, we again emphasise temperamental dominance: a person with AN who has excessive striatal-induced seeking behaviour, provoked by prolonged restraint of appetite, would still have (over time) a dominant PFC-related restraint phenotype. We do not propose that one cannot have striatal (or other reward-related responses) in conjunction with top-down restraint, rather, we suggest that it is one of the extremes of the model that will ‘win out’ to highlight the person’s temperamental dominance during a given time period. Finally, according to the model we propose, it is not only striatal responses that predict impulsive eating behaviour, but also responses in the amygdala, cerebellum and hypothalamus.

In summary, brain imaging studies support a taxonomy for a spectrum model of ED, whereby increased PFC responses to food stimuli correspond to reduced consumption of food, cognitive restraint and rigidity, obsessive ruminations about food, perfectionism and high attention to detail - symptoms that are typically observed in people with AN. Conversely, increased mesolimbic reward responses to food stimuli may correspond to greater consumption of food, impulsivity and high arousal, lack of control and aversive social situations (e.g. problems in relating positively to others), promiscuity and alcoholism – symptoms that are sometimes observed in people with BN and also those with obesity. This pattern of neural activation can easily be mapped on to the previously presented spectrum model (see Figure 1). In both extremes, an imbalanced convergence on the insula may lead to a rate-limiting defect [45] that heightens the experience of anxiety and fear, particularly for the way the body *feels* (a phobia that could perhaps be termed, *pathodysmorphia*). Finally, it must be noted that we suggest that it is the *temperamental dominance* of a particular extreme (e.g. restriction versus impulsivity) that produces phenotypic behavior, and that reduction in one dominant extreme could present a new dominance at the opposing extreme, if the secondary dominance is sufficiently activated (e.g. a new presentation of impulsivity that was previously suppressed by excessive restriction).

### Potential genetic markers underlying disordered eating behaviour

In the next section, we discuss contemporary genetic findings that may help to explain how these patterns of brain activation occur in people with ED. It must be noted that the proposed candidate genes might in part contribute to the etiology of ED, but their effects are quite small and exploratory genetic studies of ED in their infancy. Furthermore, it is most likely that complex gene-environment interactions and epigenetic effects play a greater part in the pathophysiology of ED [50]. Nevertheless, we introduce some exciting candidate genes to provoke additional debate about the role of genes in the proposed impulse control model of ED.

One of the most interesting findings in genetic studies of ED comes from a genome wide linkage test by Grice and colleagues in 2002 [60]. By screening 386 genomic markers in 192 families of ED affected patients, they were able to identify an association of a region on chromosome 1 linked to AN. This region contains, among others, the genes encoding the delta opioid receptor 1 (OPRD1) and serotonin receptor 1D (HTR1D). Single nucleotide polymorphisms (SNPs) in these genes were confirmed to be associated to AN in subsequent association studies [61,62]. Further, the candidate gene approach (i.e. an a priori selection of genetic variations related to genes proposed to be involved in EDs, based on functional specification of the gene) has produced some potential linkage to ED pathology [63]. Studies on the Val66Met polymorphism in BDNF, which is largely expressed in the mesolimbic reward region of the brain, are the most robust results in association studies on EDs to date. Through collaboration of several eating disorder clinics across Europe, large ED patient cohorts of about 1,000 patients were assembled for association studies and subsequent replication studies [64-68]. The 66Met variant leads to an amino acid exchange in the functional BDNF protein, from valine to methionine at position 66, which is denoted Val66Met. This variant is one of few for which the functional implications are known: 66Met-BDNF localizes poorly to intracellular granula compared to 66Val-BDNF. This negatively affects secretion of BDNF. Despite the large cohorts used in these studies, replication studies from other groups are somewhat more inconsistent as to this variant’s association to EDs [68-73] (Reviewed in 48). Nonetheless, the BDNF polymorphism may be linked to patterns of mesolimbic activation observed in neuroimaging studies, but the link is complex and undoubtedly incorporates many other intra- and inter-cellular, as well as environmental interactions.

**Figure 3:**
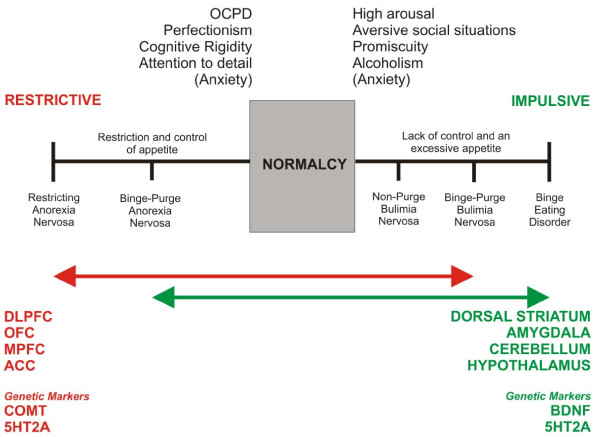
Neurobiological impulse-control model of temperamental dominance in ED.

The Val158Met polymorphism in catechol-o-methyl transferase (COMT), which was originally observed to be associated with AN in a Transmission Disequilibrium Test (TDT) performed on 51 Israeli family trios [74], and later observed to also be associated to BN [75], is of particular interest as it leads to a thermodynamically more unstable protein, which in turn leads to a lower rate of dopamine (DA) turnover in the synaptic cleft, particularly in the PFC. Subsequently, a higher DA activity in COMT-regulated neurons could be a predisposing risk factor for developing EDs. Alterations in COMT activity due to the Val158Met polymorphism is likely to have implications for DA signaling in the PFC, as clearance of DA from the synaptic cleft has been shown to be regulated via COMT in the PFC [76]. However, a recent meta-analysis of the COMT polymorphism alone and its link to AN has not shown significant results [77]. Additionally, despite set-shifting being potentially the most robust endophenotype for ED pathology [78] the COMT polymorphism alone does not relate to set-shifting impairments observed in AN and BN [79]. Both these findings suggest, as we imply in our model, that it is an interaction between genotypes (e.g. COMT vs. BDNF) that might lead to a specific ED cognitive and appetitive phenotypes (See Figure 3).

Variants related to agouti related protein (AGRP) have been associated with EDs, which is a central mediator of appetite via feeding regulating hypothalamic neuronal circuits; and SK3, a protein which regulates ion flow through the NMDA receptor [68,80-83]. AGRP acts as an antagonist on the melanocortin 4 receptor and blocks the anorexigenic effect of α-melanocyte stimulating hormone (α-MSH). Melanocortin 4 receptor antagonists are very effective in reversing different types of anorectic conditions in animal models, for example, selective melanocortin MC4 receptor blockage reduces immobilization stress-induced anorexia in rats [84].

The monoaminergic system has long been believed to be involved in the development of EDs. This is partly due to the psychiatric co-morbidities such as depression and anxiety disorders such as obsessive compulsive disorder which are commonly observed in patients diagnosed with AN. Also, clinical findings show increased CSF monoamine metabolite concentrations detected in AN patients [85]. Early findings of an association of a SNP in the promoter region of the gene encoding the 5-HT receptor 2a (5HT2A) to development of AN by Collier et al. [86], also led to this SNP being the single most studied genetic variant in genetic research in EDs. 14 studies have to date been published on this one SNP alone, 28 papers in total on association studies on SNPs related to serotonin function [63]. Despite this intense focus on variations in the gene encoding 5HT2A replication studies have been highly inconsistent in their results [63,86]. Some promising preliminary results have been published on various other SNPs associated with genes related to serotonin function such as 5HT1D [61,62], 5HT2C [87-90], the norepinephrine transporter (NET) and monoamineoxidase A (MAOA) [91]. However, these results have yet to be robustly replicated in larger cohorts.

In summary, despite there being indications that certain cognitive deficits and appetitive behaviours in ED are endophenotypic, it is not currently clear which, and how genetic polymorphisms contribute to these behaviours. Neuroimaging studies in to EDs are beginning to show consistent patterns of differential neural activation between those with and without EDs, and between the ED subtypes. However, it is not presently possible to combine genetic research with neuroimaging, because further clarification is needed about which genetic interactions (not single polymorphisms) are associated with ED pathology. Nevertheless, the proposed genes presented here are meant to provide food for thought, to encourage debate and to prompt more clarifying genetic research into EDs.

## Summary

We are proposing that neuroimaging data of people with eating disorders provide convincing evidence that supports a single *impulse control* spectrum model of eating disorders. See Figure 3. Specifically, that reduced, or at least dysregulation of striatal dopaminergic circuits, combined with varying degrees of PFC-related cognitive control contribute to the differential pathologies observed in AN, BN and BED [24]. In addition, genetic data suggests potential polymorphisms for EDs in the genes encoding BDNF, COMT and 5HT2A (48), the interactions between which may contribute to a spectrum of disordered eating. Genetic data may compliment neuroimaging findings, in that BDNF is linked to synaptic plasticity in the mesolimbic reward pathway, whereas COMT is involved in the breakdown and clearance of dopamine arriving at the PFC. Interactions between these two systems, rather than isolated polymorphisms at each gene, may contribute to ED phenotypes and the neural activation observed in neuroimaging studies, but much more work is certainly needed. We propose that it is the interaction between COMT-related PFC activity and BDNF-related mesolimbic activity that contributes to an ED phenotype along a spectrum of restrictive vs. impulsive eating behavior. An imbalance in these systems likely leads to a dysregulated orchestration of somatic and cognitive signals arriving at the insular cortex, which ultimately may cause upregulation of 5HT2A receptors and increased anxiety. Thus, it is not single polymorphisms, but complex genetic interactions (as well as gene-environment interactions and epigenetics) that likely underpins differential neural activation. It is plausible that increased anxiety is associated with activation of the amygdala and with rate-limiting defects in the insula [45]. Alterations in feeding behaviour (e.g. applying more restraint over eating in response to environmental factors) could lead to gene-environment interactions and epigenetic affects that contribute to differential functioning of these brain systems [50]. However, despite the implications of the data reviewed here, one must bear in mind that further neuroimaging studies are also needed to clarify the cognitive-emotion interactions underlying restraint vs. impulsive behaviors, e.g. by using fMRI paradigms that utilize cognitive tasks, and connectivity analysis. It is also of note that with increasing knowledge of structural differences among ED patients (e.g. using voxel-based morphometry, VBM) it will be vital to covary for structural brain differences in future brain imaging studies. Moreover, although genetic studies are convincing in this context, for ED, genetic data are still in their infancy and need further clarification. See Figure 3 for an illustration of the impulse control model of ED.

### Strengths and limitations of the model

Our model supports transdiagnostic spectrum theories of eating disorders [2,3,91,92] proposing that current diagnoses are ‘snapshots in the course of a single eating ED’ [3] and that in reality the dimensions underlying disordered eating are fluid and subject to bidirectional fluctuation across ED symptoms over the course of illness. We are proposing that it is fluctuating levels of restraint and impulsivity that underlie the presentation of a particular eating disorder at a given time point, and that it is plausible to veer between restraint and impulsivity (although *temperamental dominance* determines true phenotype over extended periods). Our model is also in line with a recent model of obesity that proposes a switch between top-down control to mesolimbic responses driving impulsive eating behaviour [93]. However, our model has some difficulty in accounting for a “mixed eating disorder” diagnosis, for those who seem to concurrently exhibit both restrictive and binge-purge symptoms, or who, for example, begin with anorectic symptoms but veer into bulimia and then EDNOS. Furthermore, the model incorporates the notion that healthy people without ED symptoms (e.g. ‘normalcy’) may exhibit, on occasion, unhealthy eating behaviors (e.g. binge eating during social gatherings, excessive dieting at the start of a new year), but without the behavior becoming pathological (e.g. on the extremes of the spectrum). Our model also provides thresholds (e.g. level of restrictive vs. impulsive eating behavior) to indicate where normal ends and pathological begins as a useful adjunct to early-stage treatment before the ED becomes ‘extreme’, and would enable EDNOS cases to be identified on a measure of ED severity based on such thresholds. Measures could adopt personality and temperament questionnaires (e.g. Restraint Scale, Impulsivity Questionnaire) rather than weight. Another important issue is that neuroimaging studies still often show controversial and inconsistent results, due to methodological and analytical differences, scanning procedures and equipment, paradigms selected s Thus, despite the excellent reviews so far on the topic of neuroimaging in ED [21-24], it is still premature to draw definite conclusions about the findings of brain imaging research.

## Conclusions

It seems that the proposals for the new ED criteria in DSM-V are beginning to embrace a transdiagnostic spectrum approach by relaxing the rigid number of binges and purges required, removing rigid diagnostic language (e.g. intense fear, refusal) and by extending the impulsive extreme of the ED spectrum by including BED. Incorporating a trans-diagnosis in the development of DSM-V can only strengthen the likelihood that ED suffers will escape EDNOS in favor of a definite diagnosis on a disordered eating behavior spectrum that likely fluctuates over a lifetime. This potential mindset shift will no doubt be, in part, fuelled by advances in neuroscientific research that will enable researchers to delve into and further clarify the neurobiological underpinnings that shape the ED mind, ready for further improvements in the DSM-VI. With the continued advancement of neuroimaging and genetic technologies, it might be possible in the future to measure at which point a person lies on this spectrum, using a simple brain scan or blood sample. Nevertheless, in the advent of the publication of the DSM-V, changes to the rigid criteria and the inclusion of a broader spectrum of EDs are a positive step in the right direction to improve diagnosis and treatment. Here we present an *impulse control* spectrum model of EDs, in order to add to the current debate on the transdiagnostic approach to diagnosis and treatment.

## Competing interests

The author(s) declare that they have no competing interests.

## Authors’ contributions

All authors (SJB, MRA, CB, HBS) contributed to the planning and writing of the manuscript, the concept was initially devised by SB and MRA. All authors read and approved the final manuscript.

## Pre-publication history

The pre-publication history for this paper can be accessed here:

http://www.biomedcentral.com/1471-244X/12/76/prepub
